# Illness experience of adults with cervical spinal cord injury in Japan: a qualitative investigation

**DOI:** 10.1186/1471-2458-13-69

**Published:** 2013-01-24

**Authors:** Ayako Ide-Okochi, Yoshihiko Yamazaki, Etsuko Tadaka, Kazumi Fujimura, Toshie Kusunaga

**Affiliations:** 1 Department of Nursing, School of Medicine, University of Ehime, Ehime, Japan; 2 School of Social Welfare, Nihon Fukushi University, Aichi, Japan; 3 Graduate School of Nursing, School of Medicine, Yokohama City University, Kanagawa, Japan; 4 Graduate School of Nursing, Osaka City University, Osaka, Japan; 5 Department of Social Welfare, Faculty of Humanity, Seitoku University, Tokyo, Japan

**Keywords:** Illness experience, Biographical reinforcement, Loss of self, Social participation, Social relationship, Spinal cord injury

## Abstract

**Background:**

There is growing recognition that healthcare policy should be guided by the illness experience from a layperson’s or insider’s perspective. One such area for exploration would include patient-centered research on traumatic Spinal Cord Injury (SCI), a condition associated with permanent physical disability requiring long-term and often complex health care. The chronicity of SCI can, in turn, affect individuals’ sense of self. Although previous research in Western countries suggests that people with SCI find a way to cope with their disability through social participation and family bonds, the process of adjustment among people with cervical SCI (CSCI) living in Japan may be different because of the restrained conditions of their social participation and the excessive burden on family caregivers. The purpose of this study was to examine the impact of injury and the process of accommodation in people with CSCI in Japan.

**Methods:**

Semi-structured home interviews were conducted with 29 participants who were recruited from a home-visit nursing care provider and three self-help groups. Interviews were recorded, transcribed and analyzed based on the grounded theory approach.

**Results:**

Five core categories emerged from the interview data: being at a loss, discrediting self by self and others, taking time in performance, restoring competency, and transcending limitations of disability. Overall, the process by which participants adjusted to and found positive meaning in their lives involved a continuous search for comfortable relationships between self, disability and society.

**Conclusions:**

The results of this study suggest that persons with CSCI do not merely have disrupted lives, but find positive meaning through meaningful interactions. Family members added to the discredit of self by making the injured person entirely dependent on them. Gaining independence from family members was the key to restoring competency in people with CSCI. At the same time, social participation was pursued for transcending the limitations of disability. The results also imply that social issues affect how people interpret their disability. These findings suggest that public health policy makers should recognize the need to enhance independence in people with disability as well as change the social assumptions about their care.

## Background

In Japan, it is estimated that more than 100,000 persons suffer from spinal cord injury (SCI), and that 5000 persons incur a SCI each year [1]. Of these persons, the proportion with cervical SCI (CSCI) is estimated at about 60% [2]. People with CSCI experience a variety of difficulties resulting from a sudden onset of disability. These difficulties, which include motor deficits, sensory deficits, and bowel and bladder dysfunction, require ongoing medical management. Therefore it is crucial that healthcare professionals understand and take heed of the layperson’s illness narratives to deliver patient-centered health care [3].

A number of authors have attempted to illustrate not only the impact of chronic illness on the lives of those experiencing it, but also how they try to make sense of their illness. Bury [4] regarded chronic illness as constituting a major disruptive experience in the past, present, and future — what he termed a ‘biographical disruption.’ Charmaz [5] developed the idea of ‘loss of self’ [5], in which people with physical disabilities experience the interaction between self and significant others as having a negative impact on their social life and self-image.

In contrast to these studies, which presented the negative impact of chronic illness on personal relationships and esteem [4,5], some studies found that the illness experience can also have positive consequences. For example, in one study, people suffering from arthritis tried to interpret the cause of illness in relation to their biography, and employed strategies to create a sense of coherence — a process Williams [6] termed ‘narrative reconstruction.’ Similarly, in a study of HIV-positive men, illness onset was associated with positive consequences which the researchers termed ‘biographical reinforcement’ [7]. Specifically, men who had lived with hemophilia and were infected with HIV through blood transfusion did not experience HIV as biographically disruptive. This was in comparison to men who were infected after practicing unprotected sex with a homosexual partner. The authors explain that the former group experienced ‘biographical reinforcement’ as they only had to modify their already adjusted life and biography as a result of their chronic condition.

A few studies have explored the positive experience of living with SCI. Based on Charmaz’s ‘loss of self’ concept [5], Yoshida [8] argued that persons with traumatic SCI experience a pendular process between the nondisabled and disabled self. Furthermore, Yoshida [8] argued that the outcomes of identity reconstruction were ‘loss,’ ‘sustainment,’ ‘integration,’ ‘continuity’ and ‘development of the self.’ Based on a phenomenological study, DeSanto-Madeya also suggested that persons with SCI and their family members positively interpreted their life with disability as a continuous process of learning which might provide a family bond and a new social goal [9].

Based on these studies, the factors that may be relevant to the achievement of successful emotional adjustment post-SCI are presented. First, it is considered that social participation is essential for the achievement of successful accommodation [8,10,11]. In particular, some authors [8,10-12] have focused on social participation through labor, because working provides opportunities not only to obtain financial security, but also to enhance self-worth through meaningful interactions in the workplace [12]. Second, it is considered that having a strong family bond helps people with SCI heal their anxiety, get to know the injury, and reconstruct their life after injury [9,12].

However, these findings may not be applicable to persons with CSCI in Japan. In terms of social participation, it has been estimated that people with a disability occupy only 1.65% of the current Japanese labor force in private companies [13]. Social participation and even just leaving the house were identified as difficult for ventilator-dependent persons with CSCI because of a lack of aides other than family caregivers [14]. With respect to the strong family bond [9], relying on caregivers for self-care may negatively influence the psychological outcomes of persons with SCI in Japan. Specifically, about 75% of family caregivers in Japan, most of whom are the parent or spouse [15], provide care for more than 19 hours per day for care recipients with SCI [14]. This is in comparison to Western studies which have reported family caregivers as providing less than 10 hours of care per day [14]. Consequently, the care burden for Japanese families is significantly higher and likely to produce restrictive consequences in the lives of people with CSCI, including less independence in decision making [16].

The goal of this study was to characterize how persons with CSCI living in Japan manage their illness, achieve a sense of control over their life, and find positive meaning and continuity from their former life despite their injury — what some authors have termed ‘accommodation’ [10,11,17]. Understanding how they have successfully adjusted to their lives and found positive consequences may hold important lessons about healthcare policies that could support people with SCI not only to survive, but also to find positive meaning in their life.

## Methods

### Grounded theory

Data were collected and analyzed based on the grounded theory approach [18]. Grounded theory involves the application of empirical methodology to the analysis of the data, and it is used for the purpose of building theory from the data. According to the grounded theory approach, theory is not developed merely on description, but also by an arrangement of categories (concepts) that are systematically interrelated to present an explanation of the phenomenon [18]. This methodology was used because this study aimed not only to describe the process of adjustment, but also to delineate the relationships between concepts found in the process of adjustment. In this study, ‘process’ refers to activities, interactions, and emotional responses related to the injury.

### Study sample and sampling strategy

The study sample was obtained through professional and personal contacts. Following written approval of the University of Tokyo’s Research and Ethics Committee, a query letter was sent to 31 eligible participants with CSCI who were interested in being interviewed. Their names and addresses were given with consent by representatives of one home-visit service provider and three self-help groups. The home-visit service provider was located in Ehime Prefecture. The three self-help groups were located in Ehime Prefecture, Hyogo Prefecture and Osaka Prefecture. Ehime Prefecture is primarily rural, Osaka Prefecture is urban and Hyogo Prefecture has elements of both. These self-help groups developed under the influence of the independent living movement that originated and developed in the US [19]. Japanese people with disability who practice independent living usually establish a Center for Independent Living, which enables them to secure the availability of personal assistants [20].

The query letter included a brief description of the study and asked for participation. In addition, the principal investigator telephoned all potential participants to explain the study purpose and schedule a mutually convenient date and time to meet. Although the process of sampling was not theoretically ideal, this study’s systematic data collection process was considered acceptable, because constant comparisons between concepts were still being made during analysis [18].

Patient demographic data are presented in Table 1. The final sample consisted of 27 men and two women who sustained a traumatic CSCI. The participants recruited from the home-visit service provider included elderly persons who incurred their injury at age 40 or older. Conversely, participants recruited from the self-help groups tended to have incurred the injury in their teens or 20s. Compared with the ratio of student participants returning to the same school (N = 7, 53.8%), it was difficult for the adult participants who were employed at the time of onset to return to the same job (N = 0).

**Table 1:** Demographic profile of the participants (N = 29)

**Characteristics**		**Number**	**Percent**
Gender	Male	27	93.1
	Female	2	6.9
Age	Mean ± SD (range)	48.1 ± 12.4 (26–77)	
Age at injury	Mean ± SD (range)	30.7 ± 16.3 (14–69)	
Duration of disability	Mean ± SD (range)	16.9 ± 9.9 (4–36)	
Level of injury	C1	2	6.9
	C3	6	20.7
	C4	10	34.5
	C5	6	20.7
	C6	3	10.3
	Unknown	2	6.9
Type of injury	Complete	17	58.6
	Incomplete	12	41.4
Cause of injury	Road traffic accident	15	51.7
	Sporting accident	9	31.0
	Fall	3	10.3
	Other accident	2	6.9
Employment at the time of onset	Employed	14	48.3
	Returned to the same job	0	
	Student	13	44.8
	Returned to the same school	7	
	Retired	2	6.9
Employment at the time of interview	Employed	10	34.5
	Unemployed	19	65.5
Marital status	Married	10	34.5
	Single (never married)	17	58.6
	Divorced	2	6.9
Household composition	With any family members	22	75.9
	Lives alone	7	24.1
Main caregiver	Family members	18	62.1
	Spouse	9	
	Mother	6	
	Father	2	
	Siblings	1	
	Paid caregivers	11	37.9
Receiving any kind of pension	Yes	26	89.7
	No	3	10.3

Seven out of 29 participants had their accidents at work and qualified for compensation. Three participants were dependent on a ventilator 24 hours a day. Seven participants could hold their torsos without a thoracic belt and five could walk with a stick or by holding a handrail. The other 22 participants could not hold their torsos. Thirteen participants could propel their wheelchair and two could drive a modified car.

### Interview

Interviews were conducted over a 5-month period (April to August 2009). The interview guide was developed around two research topics: 1) the impact of injury on participants’ lives; and 2) how participants achieved a sense of control over their life and found positive meaning and continuity from their former life despite their injury. For this purpose, the study focused on: 1) perceptions of self with CSCI; 2) performance of self-care and self-management of symptoms; and 3) conditions of social participation and social relationships.

Although the self is constructed separately through social relations, at the same time there is a whole self or identity that is an integration of various aspects of self and is consistent throughout the life cycle [8,17]. The ‘self’ concept is used in this study as an organization of various aspects of self that have reshaped after the injury and constitute identity.

All interviews, with one exception, were conducted in the participant’s home. This was an ideal location because it enabled the provision of a comfortable atmosphere and allowed the researcher to obtain data through observation [9]. Data were collected by the principal researcher in audiotaped and transcribed individual interviews. The principal researcher observed the participants’ interactions with caregivers and this data provided clues for interpreting the relationships between concepts.

### Analysis

In the grounded theory approach, analysis involves open coding, axial coding and selective coding to develop analytic categories [18]. Coding means ‘deriving and developing concepts from data’ [18] through constantly going from data to concepts.

Open coding begins with reading the transcripts several times then dividing the transcripts into codes/concepts, which are later compared with other codes/concepts derived from other participants’ transcripts in terms of characteristics and modes. Axial coding is used to interrelate codes/concepts for the purpose of making a more abstract hypothesis, which is derived from constant comparison between categories/themes. Selective coding is used to develop core variables that integrate categories into a theoretical construction.

Data were considered saturated when no further codes could be identified, the existing categories were coherent, and there were enough variations explaining the categories. Although data saturation had been achieved after the completion of 28 interviews, interviews continued until the researcher finished interviewing all of those who had already volunteered to participate in the study.

### Methodological rigor

Credibility, transferability, dependability, and confirmability are the criteria used to support the rigor of qualitative research [21]. Credibility was demonstrated through a) member checking; b) peer debriefing; c) prolonged involvement; d) persistent observation; and e) triangulation [21]. With respect to a) member checking, five participants were contacted by e-mail and were given a summary of their transcripts and of the emergent themes developed from the data of all participants, to ensure that the interpretation was truly reflective of their experience. All five participants recognized the themes as true reflections of their experience of living with CSCI. With respect to b) peer debriefing, a qualitative research expert checked that the data and interpretation were coherent and audited the study process. As for c) prolonged involvement, and d) persistent observation, the principal author became acquainted with 18 participants prior to the individual interviews and had opportunities for observational study over the 5 month duration of the study. Moreover, triangulation was ensured because data were collected through both interviews and observation. Differences in the characteristics of participant groups (recruited through home-visit service vs. through self-help groups) also demonstrated variations that contributed to the construction of an abstract explanatory frame. Transferability was accomplished by providing a thorough, dependable decision trail to demonstrate that the research findings have meaning to people in similar conditions. Dependability was ensured by providing a written decision trail of how the researcher made categories from raw data. Confirmability was accomplished by providing a thorough description of how data were collected.

### Ethical considerations

The study was conducted after approval was obtained from the Research Ethics Committee of The University of Tokyo. All participants were informed of the study objectives and design and gave their written consent for the interviews. They were free to terminate the interview if they wished.

## Results

Five main themes emerged from the data. These themes revealed the impact of CSCI on people’s lives and how they reconstruct their life after the onset of disability. The five themes were: a) being at a loss; b) discrediting self by self and others; c) taking time in performance; d) restoring competency; and e) transcending limitations of disability. These themes and their subcategories are presented in Table 2.

**Table 2:** Themes and subcategories

**Theme**	**Subcategories**
Being at a loss	Unknown body
	Unknown lives
Discrediting self by self and others	Discrediting self in physical ability and appearance
	Discrediting self in dependence
	Being perceived as incompetent by others
Taking time in performance	Taking time in accomplishment
	Taking time in reaching normalcy
Restoring competency	Finding retained abilities
	Developing independence
Transcending limitations of disability	Seeing self from a new perspective
	Setting a new social goal

### Theme 1: Being at a loss

#### Unknown body

Participants constantly experienced physical disorders that were unknown before the injury. Immediately after the injury, participants perceived that their “legs did not move” and/or that they “could not breathe.” Participants demonstrated that they could not understand how they had an “illness” that could not be cured. A participant stated, “I did not have any idea what would happen if a vertebrate animal had damage to their spinal cord.” This participant spoke of his disappointment when he could not find even a small improvement after surgery performed by physicians a year after the onset of disability.

Disorders in physiological function and mobility were mentioned as current sources of disruption. A participant spoke of his cynical perception of the body that did not obey his will. Bowel disorders bothered him even when he had become competent in managing bowel symptoms after a decade of struggle.

“I had managed my evacuation. However, in short, I came to have more bowel incontinence. On the way, things have changed dramatically and I lost control on the way.”

#### Unknown lives

Participants also spoke of their struggle in dealing with the uncertainty brought by the injury into their lives. At the time of discharge, participants and their family members who did not own a house struggled with finding an apartment. Participants and their family members were also anxious about living at home with a small number of family members taking responsibility for care.

“I am from A (prefecture). I couldn’t live even if I returned to A. That’s why I have lived in a hospital all the time.”

Participants also spoke of their uncertainty in finding a way to make a living. Unknown future financial status was stressed by participants who were not eligible for disability pensions. They were afraid of living under social security. Uncertainty about the future after their parents were deceased was also expressed by participants who were cared for by their parents.

### Theme 2: Discrediting self by self and others

#### Discrediting self in physical ability and appearance

Participants stressed their perception of incompetence when they compared themselves with their former self as an able-bodied person. Participants stated that prior to the injury, they were responsible for managing their life. However, because of the injury, participants stated they became a person who could not do anything without the aid of someone else.

Participants scornfully described their body with CSCI in terms such as ‘a disabled body on a wheelchair.’ As an example of self-discrediting, one participant who anticipated being stigmatized tried not to take part in a funeral, even though it is a social obligation for the only son to be a leader of the ceremony.

“To be honest, with this disabled body, I did not want to conduct my father’s funeral. Because my body is disabled, I hated to attend his funeral.”

#### Discrediting self in dependence

Participants suffered from feelings of being a burden on their family because they thought they chained their family to the injury and to living a restricted life to care for them. On the other hand, mothers and wives voluntarily took on their responsibilities as caregivers and in specific cases, a mother quit her job and a wife took a course toward a license in caregiving. A participant said that if his mother had not taken care of him, his parents would feel embarrassed if neighbors came to persuade them to look after their son.

“As you know, the countryside has a closed side. That’s why it would breach customs if my family did not take care of me.”

Participants themselves considered that they had to endure restricted life conditions in order to save parents’ faces, when their needs did not meet the family caregivers’ intentions. As a pattern of self-determined obedience, some participants considered that they had to postpone their plan to live independently because they knew parents wanted to take care of them as long as possible. Participants stated that living with family caregivers was stressful, because they had to take parent’s needs into consideration.

“I personally would rather have my life managed by shifts of paid attendants. As for my parents, they have a desire to be involved in my care as long as they are active.”

In addition to the conflict on the management of care service, some participants saw their relationships with family as strange and conflicting because they were taken care of by their aged and ailing mothers, while it was social obligation for a son or daughter to take care of their aged parents.

“I knew my mother had devoted her life to me. In my mind, I knew my behavior was not good. When she wants me to do something for her, I can do nothing.” 

#### Being perceived as incompetent by others

Medical professionals were considered to treat participants as incompetent persons. In some cases, explanations of prognosis and self-care methods were made to parents or a spouse, rather than to the participants themselves. Four participants had been rejected for rehabilitation opportunities because medical professionals considered that it was meaningless to admit persons “who could not even press the nursing button.” There was a participant who had to go to a long-term facility because a municipality considered that he did not have the ability to live in the community because he could not manage his toileting by himself.

“Municipality workers also did not know what to do and they told me to go to a long-term facility. I declined, saying that I wanted to manage my living by myself.”

Participants also felt that they were treated by family as a person who did not have the right to enjoy casual social interactions. In most cases, participants who lived with their family did not have the right to decide how to spend their money. In particular, husbands with injury complained that they lost their monetary control because their family members perceived them to be incompetent because of their lost physical ability to earn money, which in turn led the family members to make decisions on expenses.

“I cannot go drinking freely. I want to go to B (district). . . .My wife and my son told me, “You did a silly thing. What will you do, since you don’t earn money?”

### Theme 3: Taking time in performance

#### Taking time in accomplishment

Time was a source of frustration in participants’ everyday lives. Completing their everyday needs by themselves took hours, even for those who could propel their wheelchair. Some of the participants stated that they stopped using their own body to pursue goals, even though they were afraid of losing the physical abilities learned through rehabilitation.

“Because the time (for paid care service) is fixed, if I have to do something within the fixed time, for example, brushing my teeth, I can do it by myself, but it takes a long time.”

#### Taking time in reaching normalcy

It took decades for participants to reconstruct their life as being as normal as prior to the injury. A participant said that it took 20 years from the onset of injury until his bladder infection eased, after a urethral catheter at the time of his last hospitalization replaced intermittent catheterization with his mother’s help. Another participant spoke of his long struggle with a skin disorder that inhibited his social participation.

“When I gradually started social participation, I had pressure sores again. . . .I imagined that I could go here and there if I did not have pressure sores.”

Time was crucial for participants to get accustomed to health management and to nurture their wish for social participation. In one participant’s case, a decade had passed since he went out on casual occasions other than seeing a physician. Family members also required time to modify their perception of caregiving. It took years for family members of participants to give up their role as a main caregiver, mainly because of deteriorating health and age.

“Until then, my mother had looked after me all the time, but she got old and it’s hard for her”

### Theme 4: Restoring competency

#### Finding retained abilities

Participants found that they could reach the goal by using their own body in modified methods of self-care learned through rehabilitation. One participant found that she could still fulfill a social role as a housewife by adjusting her body to a remodeled kitchen. Another found that he could still go bowling in a wheelchair on an excursion for persons with disabilities. Praise not only from his wife, but also from the civil servants who organized the excursion made him feel more competent in his retained physical abilities.

“They (the civil servants) said, ‘you too can do it, because that person was able to do it’. When I tried to make a running start and gather speed like an able-bodied person, I couldn’t, but I could do it when I did it slowly.”

Participants found that they could achieve a goal by using someone as an alternate for their injured body. Participants did not interpret using paid caregivers negatively because it meant that they still had the intellectual ability to manage paid workers.

“I think I have to manage by using my brain instead of using my body. The goal is that I finish something. If my body doesn’t work, then I may use the attendant.”

#### Developing independence

Six participants started living independently after being torn between the necessity of being cared for and a sense of guilt in becoming a burden on family members. The experience of managing their life without family members’ support made participants competent enough to live alone after the death of their parents. Some participants also stated that independent living was necessary for having a good family relationship.

“It was a big change for me to talk with my parents equally. I had not allowed my parents to be chained to my injury. I think we can now understand each other more deeply. It is not good to discuss everything with them because I sense that I could be a constant burden.”

Participants felt that self-competency was strengthened by a family member’s positive assumptions about their management skills. A participant who had lived in the countryside described how it was pleasant to learn that his parents did not have any anxieties about him living alone, and that they boasted about him to others who had insisted on the parents taking care of him.

“I am delighted that my parents do not have any anxieties about my independent living with this body.”

### Theme 5: Transcending limitations of disability

#### Seeing self from a new perspective

Encounters with people such as those with poliomyelitis, caregivers at care facilities, and ordinary citizens obliged participants to reflectively consider the definitions of human beings and the self. It was not until participants could see their self from a new perspective that they could re-enter the nondisabled world. When talking with a hospital nurse, one of the participants said that it was not acceptance of the disability itself but the realization that he was 25 years old and should go out at his will.

“I said to a nurse, “I cannot wait to go out, but I have to ask someone to help me eat, and I have no courage to ask”. Then, the nurse said, “You are 25 years old. What does a 25-year-old adult say?””

Focusing on a new joy in life was another way for participants to see themselves and the world from a new perspective. A participant who sustained his SCI when he was 15 years old stated that his life enjoyment was initially affected not so much by his disability but by the comments of an art teacher telling him that he was short on art talent, which had made him deeply depressed for the first time since the accident occurred.

#### Setting a new social goal

Participants spoke of their will to be connected to society through labor, social networking, self-help activities or the disability rights movement. Except for two participants, who were employers, the participants’ wages were less than the amount of government-funded disability pension (*syogai kiso nenkin*). A participant who looked for open employment said that he earned only 3,000 Japanese yen (< 40 US dollars) a month for 5 to 6 hours work inputting data, until the company went into bankruptcy. Despite the low wage, he continued to look for open employment because he saw the highest value in social participation through working.

Despite limited social opportunities other than routine visits to a day care facility, a participant interpreted his life with disability positively because he could enjoy making contact that he thought the injury had provided.

“I look forward to this kind of occasion. Like you, I can talk with girls. This is also a part of my pleasure.”

For some participants, activities related to their self-help group were the priorities in their modified social goals. A participant spoke of his intention to take part in activities to support other persons with disabilities to achieve social participation.

“The one who rarely goes out should, I think, take the chance to go out, even for only 10 minutes. With that, I guess not only the person, but also the family feel refreshed.”

## Discussion

We have presented a study that focused on the lives of individuals with CSCI in Japan. This qualitative study was designed to understand the impact of CSCI on the lives of affected persons, in particular, how these persons make sense of, and adjust to their injury rather than being shaped by the disabling consequences of their SCI. In the process of life reconstruction, the negative influence of *Discrediting self in dependence* could not be eased in a short time, because it was a social obligation for both family and participants to take care of/be taken care of at home. It was not socially expected for persons with severe physical disability to live independently. However, their dependent status made some participants refrain from both being assertive with their family caregivers and being socially active. That is why, for some participants, *Developing independence* was an essential step in becoming socially reintegrated.

### Source of struggles: possible explanations and comparison with other studies

Our findings indicate that participants’ illness experience was a life-long struggle to make sense of their illness and manage their lives in spite of the injury. The theme *Being at a loss* is in keeping with Yoshida’s [8] discussion of self-reconstruction as a continuous process of adjustment and DeSanto-Madeya’s [9] presentation of the meaning of living with SCI as a continuous learning process. The theme *Taking time in performance* is similar to Corbin and Strauss’s ‘biographical time’ [17] in that persons with chronic illness or disability suffer from the fact that they might waste time because they could not accomplish the attempted necessary tasks of living because of their illness or disability.

As for the specific aspect in Japan, in the theme *Discrediting self by self and others*, the current results suggest that family members and healthcare professionals tended to cause the participants to internalize more stigma by expecting them to be entirely dependent, whereas persons with chronic health disorders in American society were expected to fulfill those responsibilities [5]. It is a matter of course for persons with CSCI to be provided care for self-management [19]. However, we assume that Japanese women’s social status as a caregiver had in part influenced the participants’ autonomy because it is a mother’s obligation to protect their disabled children, although it often results in suppression of the disabled persons’ views [22].

### Process of reconstructing self and lives: possible explanations and comparison with other studies

Developing independence was also important for some participants to restore competency, particularly those who incurred their injury at a young age. Because they were robbed of their autonomy by family caregivers, it was important for them to live separately from their family to prove their competency. Between countries, there are different reasons for conflict between family members. American family members expect persons with chronic illness to maintain independence even after the onset of the illness [5]. Conversely, Japanese family members expect persons with disabilities to entrust family members with their care, because family intentions overlay a person’s will [23]. Although these findings imply sociocultural differences in family relationships, this study agrees with a number of others that demonstrate the importance of the impacts of family relationships on the consequences of disability [9,12,22,23]. This study supports the need to continue to address the implications of changes in family relationships after the onset of disability.

In the process of developing a new concept of self in order to positively interpret the consequences of disability [5,8,9], Corbin and Strauss [17] demonstrated that some people not only reach the ‘stage’ of acceptance, but also go on to a state of ‘transcendence’. In this state, they go beyond limitations of their body through finding a real joy in living. Some of the participants may have reached this state of transcendence [17] by looking for modified social goals [10,11]. Our findings indicate that not only through labor [8], but also through various forms of social participation, can participants’ self-worth be enhanced. Taking into consideration that persons with disabilities in Japan are more house-bound [14] and social inclusion is not the norm [23], it is necessary for future research to further determine what persons with CSCI want to pursue as their long-term objective and how they can realize it.

### Implications for the role of professionals

Persons with disabilities and their families in Japan are reluctant to contact social services to obtain practical support because taking care of family by family members is the socially accepted behavior [22,23]. Instead of practical support, family caregivers of persons with disabilities expect health professionals to support them emotionally with praise [23]. We suggest that health professionals in Japan should consider both roles in supporting persons with disability: providing emotional support for family caregivers who support persons with disability, and changing the social norms of caregiving to let society accept more autonomy and independence for persons with disability.

### Limitations of this study

Because the process includes outcomes in nature [18], as Yoshida [8] confirmed as being a limitation of studies of illness experience, the relationships between process and outcome are ambiguous in this study. This study supports the need for a longitudinal analysis of impact, coping process and psychological growth after the onset of injury.

Limitations derived from the sample should also be noted. The sample in this study was more male-dominant than samples in other studies [8,9,24], which had similar gender ratios of about 4:1 to national SCI statistics. The findings of this study may therefore only be applicable to the male population. Compared with respondents in a previous study in Japan [14], the participants of this study probably had more chance to meet with healthcare professionals and paid caregivers who provided social support. The impact of financial status on the process of finding positive meaning in disability should be further explored in the Japanese population.

## Conclusions

This study focused on the illness experience of people with CSCI in a Japanese context. It was not only their suffering, but also an ongoing process in which they found continuity with the nondisabled self, and positive meaning through meaningful interactions with significant others. In particular, the impact of relationships between family members on the process of accommodation was highlighted. Social participation was important for enabling persons with CSCI to transcend the limitations of their disability. Japanese healthcare professionals can be a resource for persons with injury to find a way to cope with their disability through meaningful interactions. Healthcare policies that enable healthcare professionals to be continuously involved with persons with disability should be considered and implemented.

## Competing interests

The authors declare that they have no competing interests.

## Authors’ contributions

AI designed the study, collected and analyzed data, and drafted the manuscript. YY helped to conceive the study, participated in study design and helped to review the manuscript. ET helped to conceive the study and helped to review the manuscript. KF and TK helped to review the manuscript. All authors read and approved the final manuscript.

## Pre-publication history

The pre-publication history for this paper can be accessed here:

http://www.biomedcentral.com/1471-2458/13/69/prepub
